# Cardiac Implantable Electronic Device Infection: From an Infection Prevention Perspective

**DOI:** 10.1155/2015/357087

**Published:** 2015-10-13

**Authors:** Sangeeta Sastry, Riaz Rahman, Mohamed H. Yassin

**Affiliations:** ^1^Division of Infectious Diseases, University of Pittsburgh Medical Center (UPMC), Pittsburgh, PA, USA; ^2^Department of Internal Medicine, UPMC Mercy, Pittsburgh, PA 15238, USA; ^3^Infection Control and Hospital Epidemiology, UPMC Mercy, Pittsburgh, PA, USA

## Abstract

A cardiac implantable electronic device (CIED) is indicated for patients with severely reduced ejection fraction or with life-threatening cardiac arrhythmias. Infection related to a CIED is one of the most feared complications of this life-saving device. The rate of CIED infection has been estimated to be between 2 and 25; though evidence shows that this rate continues to rise with increasing expenditure to the patient as well as healthcare systems. Multiple risk factors have been attributed to the increased rates of CIED infection and host comorbidities as well as procedure related risks. Infection prevention efforts are being developed as defined bundles in numerous hospitals around the country given the increased morbidity and mortality from CIED related infections. This paper aims at reviewing the various infection prevention measures employed at hospitals and also highlights the areas that have relatively less established evidence for efficacy.

## 1. Introduction

Like any foreign body inserted in a patient, an implantable cardiac device can also become infected. The Centers for Disease Control and Prevention (CDC) recognizes a surgical site infection (SSI) in a clean wound as a rate of 1% or less on average [1]. It has been difficult to determine the true incidence of cardiac implantable electronic device (CIED) infection due to the lack of a comprehensive registry or mandatory reporting system for these infections. However, the rate of CIED infection has been estimated to be between 2 and 4% [2–4] with an impressive 124% and 57% rise in infection rate from the years 1990 to 1999 [5] and from 2004 to 2006 [6], respectively. This rate of CIED infection is slightly higher than that of a total knee replacement (TKR), total hip replacement (THR), or other implantable surgeries with similar wound classification and given the increasing number of cardiac devices being implanted over the years as evidenced by a nationwide sample of all CIED placement procedures reporting an increase in CIED insertion from less than 200,000 devices per year in 1993 to close to 350,000 devices per year in 2008 [7], we do believe that the actual rates of CIED infection may be higher than what is being recorded.

Multiple risk factors for the occurrence of CIED infection have been described in the literature. Older patients with multiple comorbidities such as congestive heart failure (CHF), metastatic malignancy, corticosteroid therapy [8], and those with renal failure [9] are more likely to develop CIED infections which subsequently increases mortality. Also, other risk factors related to the procedure itself such as the implantation of multiple leads versus a single lead, an emergency versus an elective procedure, inpatient versus outpatient procedure, and longer versus shorter procedures have been shown to increase the rate of CIED infections. Procedures done for the revision of CIED have had contradictory reports as being a risk factor for CIED related infections [10–12].

Along with the increasing rates of infection, there is concern for the rising cost of managing these infections. The in-hospital charge for CIED infection has been estimated to be about $75,000 in 1993 which almost doubled to $146,000 in 2008, accounting for an increase of about 47% per decade. Similarly, inpatient mortality associated with CIED infection has increased from 2.91% in 1993 to 4.69% in 2008, representing an increase of 1% in mortality per decade [2, 3].

Additionally, in August 2012, the Centers for Medicare and Medicaid Services (CMS) published the Inpatient Prospective Payment System and Fiscal Year 2013 Rates—Final Rule [13], which added SSI after CIED implantation as a hospital-acquired condition (HAI). As such, the CMS considers these infections to be reasonably preventable and per the ruling, hospitals are deemed to be no longer eligible for payment from CMS for treating these infections if such a complication should arise, thus increasing the need to define appropriate infection control measures for the prevention of CIED related infections.

## 2. Infection Prevention Measures

Despite the reports on the increasing incidence and significant mortality related to CIED infections, there is limited data describing the appropriate sterile techniques to be employed in the cardiac catheterization laboratory for prevention of these infections. The Laboratory Performance Standards Committee of the Society for Cardiovascular Angiography and Interventions (SCAI) published the first guidelines for infection control in the cardiac catheterization laboratory in 1992 [14] describing the utility of appropriate sterile techniques in the cardiac catheterization laboratory. Also, the Surgical Care Improvement Program (SCIP) interested in improving surgical care by significantly reducing surgical complications launched a joint work program between CMS and the Centers for Disease Control and Prevention (CDC) in 2006 [15] with particular focus on infection prevention measures and an aim to reduce surgical morbidity and mortality by 25%, by year 2010. More recently, a prospective trial showed that the implementation of a comprehensive infection control program resulted in a significant reduction in the rates of CIED related infections [16], thus prompting us to review the various infection prevention measures employed at hospitals, including the measures that have relatively less established evidence for efficacy. The multiple infection prevention measures have been reviewed below and also outlined in Box 1.

### 2.1. Modification of Patient Risk Factors prior to Procedure

It is well known that smoking increases the risk for stroke and acute myocardial infarction, but it has also been implicated as a risk factor for SSIs as well as other complications related to surgical procedures [17]. Hence, smoking cessation should be advised for all patients [18], irrespective of their need to undergo implantation of a CIED. Sugar control is an integral part of the SCIP measures that recommend a blood glucose level of less than 180 mg/dL as a postoperative morning reading [19] to reduce surgical complications, including infections, and ultimately improve surgical care. Glycosylated hemoglobin of less than 7% is also recommended prior to surgery [20]. The presence of an active infection prior to the placement of a CIED may increase the risk for CIED infection and hence should be treated prior to the procedure. Currently, there is no recommendation to routinely test and treat for asymptomatic bacteriuria prior to a CIED implantation procedure. Leukocytosis of an unclear source is not a contraindication for CIED placement, especially with no evidence of any localized source of infection or bacteremia. Risk factor modification of other factors that increase the risk for SSI and perioperative complications such as CHF, anemia requiring blood transfusions [21, 22], and nutritional status should also be maximized prior to the procedure, to not only reduce infection but also reduce readmission and prolonged hospital stays.

### 2.2. Hair Removal

The CDC strongly recommends that hair should not be removed preoperatively unless the hair at or around the incision site will interfere with the operation [23]. If hair removal is required, it should be done using clippers as close to the surgical time as possible. This should not be done in the procedure suite so as to avoid contamination of the surgical field by hair via air currents1.

### 2.3. Skin Preparation

Since the microbiome found on the patient's skin can be a significant source for contamination of a surgical wound, the optimization of preoperative skin antiseptic measures is known to decrease postoperative infections. The ideal skin antiseptic agent should kill all bacteria, fungi, viruses, protozoa, and spores on the skin. It should also be nontoxic, hypoallergenic, nonabsorbable, and safe for repeated use [24]. There are numerous options available commercially; however, the three most extensively used skin preparation agents are chlorhexidine gluconate (CHG), povidone-iodine/iodine povacrylex, and isopropyl alcohol. A randomized controlled study of patients undergoing clean-contaminated surgery (abdominal, thoracic, gynecological, and urological procedures) that specifically looked at CHG-alcohol preparations versus povidone-iodine for surgical site antisepsis found that the overall rate of surgical site infection was lower (9.5% versus 16%, *p* = 0.004) in the CHG-alcohol group which was also found to be significantly more protective against both superficial and deep incisional infections within 30 days after surgery when compared to the povidone-iodine group [25]. Repeated skin cleansing is more effective than single intense scrubbing. It allows the disinfectant to extend deeper into the skin and disinfect skin appendages such as hair follicles. Using CHG wipes in the preoperative holding area has also been shown to be effective in reducing superficial and deep SSI by various randomized controlled trials conducted all over the world [26–29] suggesting the use of CHG as the preferred agent for skin decontamination preoperatively. In our institution, all patients are being offered CHG for bathing daily for 5 days prior to an elective outpatient CIED implantation procedure. Typically, inpatient procedures are emergent; hence, CHG bathing is offered on the day of admission and then continued daily for all inpatients, pre- and postoperatively.

### 2.4. MRSA Surveillance and Decolonization

Methicillin sensitive* Staphylococcus aureus *(MSSA) as well as methicillin resistant* Staphylococcus aureus *(MRSA) colonization is a known risk factor for SSI [30]. However, the actual benefit from MSSA or MRSA surveillance and decolonization preoperatively remains controversial. The use of mupirocin alone for decolonization has not been shown to reduce the rate of SSI [31] despite the reported reduction of bacteremia in hemodialysis patients and other SSIs in patients undergoing cardiac surgery [32, 33]. Decolonization with CHG, on the other hand, has been shown to be effective in reducing hospital-associated infections (HAIs) [34], including SSIs, and especially infections caused by multidrug resistant organisms (MDRO). A large randomized study and a recent meta-analysis have also shown significant benefit of surveillance and decolonization [35, 36], thereby encouraging their use.

However, according to a recent survey [37], screening for MSSA or MRSA colonization preoperatively has not been a consistent practice nationwide despite the latest guidelines from the Society of Healthcare Epidemiology of America (SHEA) endorsing a level II recommendation for* Staphylococcus aureus *surveillance and decolonization [38]. Hence, efforts should be made to employ screening prior to placement or revision of a CIED and also to begin decolonization as soon as possible, prior to the procedure.

### 2.5. Preoperative Antibiotics

Preoperative antibiotics are administered to target the organisms most likely to present on the skin and skin structures, such as* Staphylococcus aureus* and coagulase negative staphylococci and streptococci [39] and an effective preoperative antibiotic should be given at a therapeutic dose and within an appropriate time period prior to incision, to ensure adequate tissue and organ concentrations during surgery [40]. A meta-analysis of seven randomized trials suggested that antibiotic prophylaxis given at the time of permanent pacemaker insertion significantly reduced the infection rate, though the individual trials were small with an assortment of penicillin and cephalosporin regimens and yielded inconsistent results [41]. However, the overall finding that systemic perioperative antibiotic prophylaxis was beneficial is consistent with the results of two case-control studies [42, 43], a large, prospective registry [44], and a retrospective population-based study [45]. Cefazolin is the preferred preoperative antibiotic, endorsed by several guidelines for the majority of surgical procedures, owing to its established safety, favorable cost, and narrow spectrum of activity [39, 40]. Preoperative dosing recommendations for intravenous cefazolin are based on weight such that adult patients weighing <80 kg and >80 kg receive 1 and 2 gram, respectively, with 2 g recommended for the morbidly obese as well [46, 47] to be given within 60 minutes of surgical incision. Patients with history of penicillin intolerance manifesting as an uncomplicated skin rash may be treated with a cephalosporin. Allergic cross-reactions between penicillin and cephalosporins are infrequent except in patients with severe IgE-mediated reactions to penicillin; in that case cephalosporins should be avoided. Vancomycin, as a 15 mg/kg single intravenous dose [48], could be used as an alternative to cephalosporins in severe penicillin allergy. It can also be used in addition to cefazolin in cases of a previously known MRSA infection or colonization. The SCIP national initiative has stated that antibiotics should be stopped within 24 hours of all surgeries with the exception of cardiac surgery (within 48 hours).

Topical antibiotics have been tried in different implant-related surgeries (spine and foot surgery) to prevent SSI with some success but have not been studied in the prevention of CIED infections. A prospective randomized placebo controlled single centered trial in Pennsylvania attempted to provide direction by enrolling 1008 patients and randomizing them to four groups for local application of topical povidone iodine solution, neomycin, sterile nonadherent pad, and a placebo formulation, respectively, after closure of the surgical site while receiving standard systemic antibiotic prophylaxis as well and is followed for 12 months. The use of topical antibiotics after closure did not show a statistically significant benefit in prevention of postprocedural infection [49]. However, a meta-analysis of 9 retrospective studies showed that vancomycin powder in the operative wound may be protective against SSI [50] in neurosurgery and more recently CIED implantation with an antibiotic envelope has shown some promising results in an attempt to reduce SSI [51].

### 2.6. Sterile Barrier Precautions

Inside the cardiac electrophysiology and catheterization suite, sterile barrier precautions must be enforced. These precautions are similar to the standard barrier precautions recommended by the CDC for the insertion of central or peripheral venous catheters or guide wire exchange which includes the use of a cap, mask, sterile gown, sterile gloves, and a large surgical drape [52, 53]. There are no individual trials that assessed the effect of the individual items of this bundle but certainly this bundle in total has been shown to reduce surgical site and central line associated blood stream infection (CLABSI).

### 2.7. Technical Procedure Issues

Various risk factors related to the cardiac procedure itself may predispose patients to develop a CIED related infection. Data shows that use of temporary pacing prior to the implantation procedure 4, an early pocket reexploration [51], the presence of more than two pacing leads [42, 54], and the implantation of a defibrillator more than a pacemaker [55] are some of the procedural characteristics associated with a higher infection rate. Procedures done for the revision of CIED have had contradictory reports as being a risk factor for CIED related infections [10–12]. However, an antibacterial envelope for CIED implantation has been suggested to be protective against CIED infection especially in high risk patients in a small retrospective industry sponsored trial [51] as stated in the prior section.

### 2.8. Postoperative Wound Care

Appropriate wound care after the implantation of a CIED is essential to prevent contamination of the surgical site. The use of a sterile gauze or transparent semipermeable dressing and not nonpermeable plastic dressings to cover the wound is recommended [56]. Topical antibiotic ointment or creams on insertion sites are discouraged as they are generally ineffective in promoting wound healing or preventing infections and also they have the potential to promote dermatitis and antimicrobial resistance [57–59]. Dressings should be changed regularly [60]. It is prudent to monitor surgical sites visually when changing the dressing or by palpation through an intact dressing on a regular basis, depending on the clinical situation of the individual patient. If patients have tenderness at the insertion site, fever without obvious source, or other manifestations suggesting local or bloodstream infection, the dressing should be removed to allow for thorough examination of the site. Postoperative wound healing and adequate care are essential in prevention of SSI [61].

### 2.9. Scrubbing and Hand Hygiene

Adherence to proper hand hygiene practice is the most effective and least expensive way to prevent health care-associated infections (HAI), including CIED related infections [62]. The guidelines put forth by the Institute for Clinical Systems Improvement (ICSI) from the United States recommend that artificial nails should not be worn by surgical staff; all jewelry should be removed prior to beginning surgical hand preparation and to wash hands with soap and water with mechanical friction for 15 seconds if the hands are soiled or to use a waterless alcohol preparation if they are not soiled [63] prior to a surgical procedure. An Italian study reported that the* in vivo *efficacy of an alcohol-based hand rub was sustained for a period of at least 3 hours [64]. The World Health Organization (WHO) recommends scrubbing hands and forearms for the length of the time recommended by the manufacturer, typically 2–5 minutes, and allowing the hands and forearms to dry thoroughly prior to donning sterile gloves.

### 2.10. Attire

Although most SSIs are caused by the patient's endogenous flora, operating-room personnel are also a source of bacterial contamination [65, 66]. Surgical attire aims at providing a functional barrier between the surgical team and the patient. As per the standard attire in a surgical suite including cardiac catheterization labs and in compliance with Universal Precautions to reduce exposures to blood borne pathogens, guidelines recommend that the operator must don caps, eye protection gear, masks, and nonporous gowns while performing the procedure. The true effect on the prevention of CIED infection is unclear; however, since the risk of using these precautions is nonexistent [67], the recommendation is that it should be strongly considered. Shoe covers are required by the Occupational Safety and Health Administration (OSHA) in order to reduce contamination of other areas of the healthcare facility (e.g., room to room transmission). Technicians, nurses, and any other personnel should wear scrub suits, cap mask, and gloves when they assist within the sterile field of the procedure [68] as well.

### 2.11. Traffic

After the commencement of an implantation procedure of a CIED, it is important to keep all the doors to the suite closed except as necessary for passage of equipment, personnel, and the patient. Young and O'Regan conducted a prospective cross-sectional study observing the number of door openings in forty-six consecutive cardiac operations. They found a trend toward an increased frequency of door openings for patients who developed SSI (mean door openings of 94) when compared to those who did not develop infection (mean door openings of 76.4); however, the difference was not significant [69]. A correlation between the number of operating-room door openings and increased colony forming units (CFU) of bacteria in the operating room has also been demonstrated [66] but there is no evidence in the current literature that has identified a clear correlation between room traffic and rates of SSI. However, certainly keeping the traffic to a minimum is essential to keep air flow stable and avoid contamination of the surgical field [70].

### 2.12. Physical Operation Room Environment

Normothermia in the perioperative area (35.5°C or higher) is important to reduce operation complications. Hypothermia decreases neutrophil function and causes vasoconstriction that further decreases oxygen delivery. Multiple randomized controlled trials have showed benefit in reduction of SSI in keeping normothermia for both intraoperative and perioperative periods [71–73].

Supplemental oxygen in the immediate postoperative period, in addition to adequate oxygenation intra-operatively, is protective against SSI. Typically, patients undergoing CIED implantation procedures are not intubated but are likely to have CHF and it is very important to have appropriate oxygen saturation [74, 75] in the perioperative period.

Air quality and exchange requirements for CIED placement are similar to that of a standard operation room with positive pressure ventilation of at least 20 exchanges per hour with at least four of these exchanges, from the outside air. The air should pass through two filters with 30% and 90% efficiency. The airflow should be unidirectional, downwards, with an* average velocity *of the 25 to 35 cfm/ft^2^ (127 L/s/m^2^ to 178 L/s/m^2^) [76].

Operation room equipment and surface cleaning with disinfection should be performed regularly.

The catheterization lab should be treated exactly like an operation room. It should be cleaned between patients and terminally at the end of the day. The area should be clean with no visible dirt. All equipment should be cleaned and wiped at least daily with United States Environmental Protection Agency (EPA) approved disinfectant. All surgical tools should be sterilized. Flash sterilization should not be performed for any implantable devices. Sterilization should be checked using physical, chemical, and biological monitors.

### 2.13. Waste Disposal

Single use disposable catheters are the current standard for the majority of equipment utilized in the cardiac suite. Standard techniques should be employed to ensure proper sterilization of equipment that is reused. Reuse of equipment should be limited to only those that are currently permitted by the federal regulations [77]. Blood contaminated drapes, gowns, gloves, and sponges should be discarded in special containers and labelled as healthcare waste. Needles and blades should be placed in puncture proof containers [78].

### 2.14. Infection Control Education and Audits

SSI prevention is a team effort and the infection control enthusiasts of hospitals should lead in the education and training of healthcare workers in order to prevent SSI. The National Health Safety Network (NHSN) recognizes an infection related to CIED placement as SSI if it occurs within 90 days of implantation. Routine audits and rounds should be performed on a regular basis, preferably with a multidisciplinary team to ensure adherence to infection prevention measures. Surveillance of CIED related infections whether they occur as an inpatient or outpatient should be done manually or electronically and an investigation should be conducted with any increase in incidence of SSI or outbreak in the hospital.

In conclusion, we believe that the infection prevention practices for CIED implantation should receive similar attention as total knee and hip surgery in the preoperative, operative, and postoperative stages especially since these infections are associated with not only an increased length of stay in the hospital but more importantly an increased mortality.

## Figures and Tables

**Box 1:**
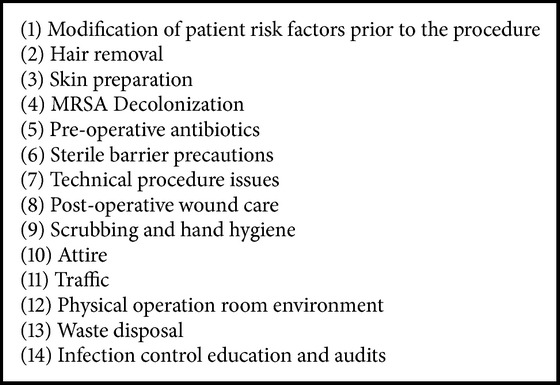
**Box 1: **Infection prevention measures for the prevention of cardiac implantable electronic device (CIED) infection.
